# miRNA408 from *Camellia japonica* L. Mediates Cross-Kingdom Regulation in Human Skin Recovery

**DOI:** 10.3390/biom15081108

**Published:** 2025-08-01

**Authors:** Soll Jin, Jae-Goo Kim, Hye Jin Kim, Ji Young Kim, Sang Hoon Kim, Hee Cheol Kang, Mi Jung Kim

**Affiliations:** 1 Human & Microbiome Communicating Laboratory, GFC Life Science Co., Ltd., Hwaseong 18471, Republic of Korea; s.jin@gfcos.co.kr (S.J.); jg.kim@gfcos.co.kr (J.-G.K.);; 2 Department of Biology, Kyung Hee University, Seoul 02447, Republic of Korea

**Keywords:** extracellular vesicles, callus, *Camellia japonica* L., microRNA, miR408

## Abstract

Wound healing is a complex and dynamic process involving several stages of tissue repair. This study has shown that extracellular vesicles (EVs) derived from the callus of *Camellia japonica* L. and their associated microRNAs (miRNAs) possess significant wound healing activities. In human fibroblasts, EVs from *C. japonica* L. stimulated wound healing and upregulated collagen gene expression. The EVs also decreased inflammation levels in human keratinocytes, supporting wound healing. Among the miRNAs identified, miR408, one of the abundant miRNAs in the EVs, also showed similar wound healing efficacy. These findings suggest that both EVs and miR408 from the callus of *C. japonica* L. play a pivotal role in promoting wound healing. Additionally, this study shows that the regulation of miRNAs between different kingdoms can be achieved and suggests a new direction for the utilization of plant-derived components.

## 1. Introduction

In recent years, interest in plant-derived substances as potential therapeutic agents has grown, particularly in extracellular vesicles (EVs) [1]. Furthermore, exosomes, a subtype of EVs, have gained attention due to their ability to mediate intercellular communication by transferring various bioactive molecules, including proteins, lipids, microRNAs (miRNAs), messenger RNA (mRNA), DNA, and other nucleic acids. These nanometer-sized vesicles, typically ranging from 50 to 200 nm, are generated within cells and secreted into extracellular space. They possess a stable structure characterized by a phospholipid bilayer and play a crucial role in cellular signaling by fusing with recipient cells and delivering their contents [2]. Recent studies have highlighted the therapeutic potential of plant-derived vesicles (PDVs), which exhibit bioactive properties that contribute to the treatment of various diseases and enhance human health. Thus, recognition of the value of PDVs in the development of novel therapeutic and cosmetic applications is growing [3].

miRNAs are short non-coding RNA molecules that play a critical role in regulating gene expression by interacting with messenger RNA (mRNA). By binding to the target mRNA, miRNAs inhibit gene translation or destabilize the mRNA, thereby modulating protein expression levels [4]. Typically transcribed within cells, miRNAs specifically bind to mRNA that encodes certain proteins, resulting in translational repression or mRNA degradation, which, in turn, suppresses protein synthesis. Regulatory functions of miRNAs are essential for maintaining normal cellular functions and supporting growth and development. However, the dysregulation of miRNA activity has been implicated in various diseases, particularly cancer [5,6]. In recent years, miRNAs have emerged as valuable biomarkers of various diseases. Their altered expression patterns, which often change in response to disease conditions, make them promising diagnostic and prognostic tools for biomedical research. A biomarker is an objective indicator of biological or pathogenic processes or responses to therapeutic interventions. The potential of miRNAs as biomarkers was first recognized when Lawrie et al. (2008) demonstrated their utility in detecting diffuse large B-cell lymphoma in patient serum samples [7]. Since then, miRNAs have been extensively studied and cited in the literature for their potential application as biomarkers of numerous diseases, supporting their relevance in advancing diagnostic precision and therapeutic monitoring.

Similarly to animals, plant miRNAs also function as post-transcriptional regulators. Current research on plant miRNAs spans various aspects of plant biology, including biological processes, growth, development, immune responses, stress adaptation, and the regulation of gene expression [2,8]. Plant miRNAs play a crucial role in plant development and growth, particularly in regulating processes, including cell proliferation, differentiation, and tissue formation in the roots, stems, and leaves. Additionally, plant miRNAs are central to plant environmental stress responses. These miRNAs enable plants to adapt by regulating gene expression in response to factors, such as climate change, heat, drought, and salinity, which are critical for their survival and ecological functions [9]. miRNA408 (miR408), identified in plants, is known for its significant physiological functions across various plant species. Predominantly expressed in chloroplasts, miR408 regulates several key processes, including photosynthesis and chlorophyll biosynthesis, processes essential for plants to maintain optimal photosynthetic efficiency, especially under stress conditions induced by high-intensity light. These processes enable plants to mitigate damage from environmental stressors, enhancing their photosynthetic resilience [10]. Apart from photosynthesis, miR408 is actively involved in plant responses to other environmental stresses, such as extreme heat. miR408 regulates the expression of specific genes that help plants activate survival mechanisms, increasing their stress tolerance and adaptability to challenging environments. These attributes have made exploring the potential effects of miR408 beyond plant biology an emerging area of interest.

Despite the well-established roles of miRNAs in plants, research on the interactions between plant-derived miRNAs from EVs and human skin cells is limited. Hence, this study aimed to explore the therapeutic potential of these plant-derived miRNAs from EVs for skin health applications in skin health. Understanding the interactions between plant-derived miR408 from EVs and human skin cells may offer novel insights into the molecular pathways associated with skin aging [11]. The discovery of cross-kingdom regulatory mechanisms mediated by miR408 may pave the way for therapeutic applications and innovative skincare strategies that utilize plant-derived miRNAs from EVs [12].

In this study, we isolated extracellular vesicles from the callus of *Camellia japonica* L. (Cj-callus EVs) and investigated their in vitro biological effects on human skin cells, with a focus on wound healing and anti-inflammatory properties. Small RNA sequencing revealed that miR408 was highly expressed in Cj-callus EVs. Subsequent experiments involved the transfection of human skin cells with miR408 and assessing various biological markers to determine their impact on skin cell functions. This study suggests the involvement of plant-derived miR408 from Cj-callus EVs in the promotion of wound healing and modulation of inflammatory processes in human skin cells. These findings suggest potential implications for future research and development in the field of skin care.

## 2. Materials and Methods

### 2.1. Callus Induction of Camellia japonica L. and Suspension Culture

For the induction of callus from the leaves of *Camellia japonica* L. (Cj-callus), leaves were surface sterilized by soaking in 95% ethanol for 60 s, followed by treatment with a disinfectant solution (50% bleach + 0.1% Tween-20) for 20 min. The sterilized leaves were washed at least three times with sterile distilled water, supplemented with 500 mg/L of cefotaxime sodium (Duchefa, Haarlem, The Netherlands) to prevent contamination from soil pathogens. Sterilized leaves were cut into 5 × 5 mm pieces and placed on a Murashige and Skoog (MS) medium containing 3% (*w*/*v*) sucrose and 0.35% (*w*/*v*) gelatin, along with 0.5 mg/L of 2,4-dichlorophenoxyacetic acid (2,4-D, Duchefa, Haarlem, The Netherlands), which was used as a callus induction medium (CIM). The cultures were maintained in the dark to promote callus formation. The induced callus was subcultured every 4 weeks for 2–3 times on a solid medium. Subsequently, the callus was transferred to liquid medium and cultured under suspension conditions for 3–4 weeks at a shaker speed of 100–300 rpm to promote further growth.

### 2.2. Isolation of Extracellular Vesicles (EVs) from C. japonica L.

EVs from *C. japonica* callus (Cj-callus EVs) were isolated using a liquid suspension culture system. Four-week-old Cj-callus cultures from a bioreactor were first separated into callus clusters and conditioned medium using 100- and 300-mesh filters. The medium was then centrifuged at 3000–4000× *g* for 20 min to remove cell debris, fibers, and large particles. Only the supernatant was collected, and this step was repeated to ensure the removal of any residual impurities. The cleared supernatant was further subjected to ultracentrifugation at 100,000–150,000× *g* for 2 h using an ultracentrifuge (Hitachi, Tokyo, Japan), which allowed EVs to settle into the pellet. The supernatants were carefully discarded, and the EV pellet was resuspended in a small volume of deionized water (DIW). The EV suspension was filtered through a 0.22 μm syringe filter (Advantec, Tokyo, Japan) for sterilization and stored at −80 °C until further use. The size distribution and the particle concentration of EVs were analyzed using Nanoparticle Tracking Analysis (NTA), and the zeta potential of the EVs was measured using a ZetaView (Particle Metrix, Inning am Ammersee, Starnberg, Germany).

### 2.3. Transmission Electron Microscope (TEM) Imaging

EVs were placed onto carbon-coated grids (EMS, Houston, TX, USA) at room temperature for 3–5 min. Excess sample was carefully removed, and the grids were allowed to air dry. The grids were then stained with 2% uranyl acetate solution (EMS, Houston, TX, USA) at room temperature for less than 10 s. Nanoparticle imaging was conducted using a transmission electron microscope (JEOL, Tokyo, Japan) [13].

### 2.4. Western Blot Analysis

Protein was extracted from EVs using RIPA buffer and quantified with the Bradford reagent (Thermo Fisher Scientific, Waltham, MA, USA). Equal amounts of protein were separated on SDS-polyacrylamide gels and transferred onto polyvinylidene fluoride (PVDF, Invitrogen, Carlsbad, CA, USA) membranes. Transferred membranes were blocked with 5% BSA and then incubated with the following primary antibodies: anti-TET8 (PHY1490A, PhytoAB, San Jose, CA, USA) or anti-PEN1 (CSB-PA875527XA01DOA, Cusabio, Houston, TX, USA) overnight at 4 °C. Following washing with TBST, membranes were incubated with horseradish peroxidase (HRP)-conjugated secondary antibody (PHY6000, PhytoAB, San Jose, CA, USA) for 1 h at room temperature. The membranes were developed using ECL (Bio-Rad, Hercules, CA, USA) on a chemiluminescence imaging system (Bio-Rad, Hercules, CA, USA) [14].

### 2.5. Cell Culture

The human keratinocyte (HaCaT) cell line and human fibroblast (HFF) cell line were cultured in Dulbecco’s modified Eagle medium (DMEM, Welgene, Gyeongsan, Gyeongbuk, Republic of Korea), supplemented with 10% fetal bovine serum (FBS) (Gibco, Grand Island, NY, USA) and 1% of a penicillin–streptomycin solution (Gibco, Grand Island, NY, USA). Cells were maintained at 37 °C in a humidified incubator with 5% CO_2_.

### 2.6. Cell Viability Assay

HaCaT and HFF cells were seeded at a density of 1.0 × 10^4^ cells/well in 96-well plates and cultured for 24 h. Cultured cells were treated with EVs at specified concentrations or transfected with a microRNA mimic (RNA double-strand oligonucleotides) for each experiment and incubated for 48 h. Cell viability was assessed using the WST-1 cell proliferation and cytotoxicity detection kit (DoGENBIO, Seoul, Republic of Korea) following the manufacturer’s protocol. After adding the WST-1 reagent, the mixture was incubated for 2 h, and absorbance was then measured at 450 nm using a microplate reader (Bio Tek, Shoreline, WA, USA).

### 2.7. Extracellular Vesicles Uptake Assay

HaCaT cells were seeded at a density of 1.0 × 10^5^ cells/well in black 24-well plates and cultured for 24 h. EVs were labeled with LipidyeII (Funakoshi, Morioka, Japan) according to the manufacturer’s protocol. Hoechst 33342 was applied to cultured cells for nuclear counterstaining, and subsequently, the labeled EVs were added to each well, followed by an additional incubation for 6 h. After treatment, the wells were washed 3 times with DPBS to remove any unbound EVs in the medium, and cells were fixed in 4% paraformaldehyde (PFA) solution for 15 min at room temperature. Fluorescence images were obtained using a fluorescence microscope (Ts2, Nikon, Tokyo, Japan) to assess EV uptake [15].

### 2.8. In Vitro Wound Healing Assay

HFF cells were seeded into a 24-well plate at a density of 5.0 × 10^4^ cells/well using Wound Healing Insert (Cellbiolabs, San Diego, CA, USA) and allowed to adhere and culture for 24 h. Following incubation, the inserts were carefully removed, and the wells were washed with DPBS to remove debris and non-adherent cells. The cells were then treated with EVs at an optimized concentration in a serum-free medium for an additional 48 h. The size of the cell-free gaps was visualized and detected under a microscope equipped with a digital camera (Nikon, Tokyo, Japan) and measured using Image J software v 1.54g. It was expressed as a percentage of the control interval.

### 2.9. Immunocytochemistry (ICC)

HFF cells were seeded at 2.0 × 10^5^ cells/well in 24-well plates and cultured for 24 h. After the initial culture, the cells were exposed to 1.5 J/cm^2^ UVA radiation using a UV illuminator (Bioteck, Seoul, Republic of Korea). Following UVA exposure, cells were either treated with EVs or transfected with a microRNA mimic, as specified for each experimental condition, and then incubated for an additional 48 h. After incubation, supernatants were discarded, and the cells were gently washed with DPBS before fixation in 4% (*w*/*v*) paraformaldehyde (PFA) for 20 min at room temperature. Cells were washed 3 times in DPBS, permeabilized with 0.1% Triton-X 100 in DPBS for 20 min, and washed again 3 times. Subsequently, a 5% bovine serum albumin (BSA, BOVOGEN, Keilor East, VIC, Australia) in DPBS for blocking solution was applied to prevent non-specific antibody binding and then incubated with collagen type I A1 (COL1A1) primary antibody (ab316222, Abcam, Cambridge, UK) overnight at 4 °C. The next day, cells were incubated with goat anti-rabbit IgG antibody conjugated to Alexa Fluor 488 (ab150077, Abcam, Cambridge, UK) as a secondary antibody for 1 h at room temperature, followed by staining with 1 μg/mL DAPI for 15 min. Finally, cells were washed with DPBS, and the cell images were taken using a fluorescence microscope [16].

### 2.10. ELISA

HaCaT cells were seeded at a density of 5.0 × 10^4^ cells/well in 24-well plates and cultured for 24 h. Following initial culture, the medium was replaced with a fresh medium containing EVs or miRNA mimics at varying concentrations, and cells were incubated for an additional 24 h. After incubation, supernatants were collected and centrifuged, then stored at −20 °C until further use. IL-6 expression levels in the supernatants were quantified using a human IL-6 ELISA kit (#430501, BioLegend, San Diego, CA, USA) according to the manufacturer’s instructions. Absorbance was measured at 450 nm to determine IL-6 concentrations.

### 2.11. qRT-PCR (Quantitative Reverse-Transcription–PCR)

Each cell line was seeded in a 24-well plate at a density of 1.0 × 10^5^ cells/well and cultured at 37 °C with 5% CO_2_ for 24 h. After incubation, the medium was discarded, and cells were washed with DPBS. Cells were then treated with appropriately diluted EVs in serum-free medium and incubated for another 24 h. Total RNA was isolated using the NucleoSpin RNA kit (Macherey-Nagel, Düren, Germany) according to the manufacturer’s instructions, and RNA quantity was assessed using a Nanodrop ND-1000 spectrophotometer(Thermo Fisher Scientific, Waltham, MA, USA). cDNA was synthesized using a cDNA synthesis kit (GenDEPOT, Katy, TX, USA) following the manufacturer’s protocol. Target gene expression was detected with SYBR Green Supermix (Bio-Rad, Hercules, CA, USA) using a real-time PCR machine (Bio-Rad, Hercules, CA, USA). Expression levels were normalized to β-actin as an internal control, and relative gene expression was calculated. Primer sequences used in this study are listed in Supplementary Table S2.

### 2.12. Small RNA Isolation

Small RNA was isolated from Cj-callus and Cj-callus EVs using a XENOPURE Plant Small RNA Purification kit (Xenohelix, Incheon, Republic of Korea) and XENO-EVARI Kit (Xenohelix, Incheon, Republic of Korea), respectively, following the manufacturer’s instructions. Purified RNA samples were further cleaned using the XENOPURE Small RNA clean-up kit, as instructed. Isolated RNA was eluted in 20 μL of RNase-free water and stored at −80 °C until further use. The concentration of RNA was quantified with a nanodrop spectrophotometer (Thermo Fisher Scientific, Waltham, MA, USA) and analyzed for quality and quantity using the 2100 Bioanalyzer (Agilent^®^ Technologies, Santa Clara, CA, USA) with the RNA 6000 Pico chip. All RNA samples were stored at −80 °C until subsequent analysis.

### 2.13. Small RNA Library Construction and Analysis

Small RNA libraries were generated using the XENO-LIBERA library kit (Xenohelix, Incheon, Republic of Korea) according to the manufacturer’s instructions. Sequencing was conducted on an Illumina NextSeq 500 platform with a 76-cycle single-end read configuration. The quality of raw miRNA-seq data was assessed using FastQC v0.11.9 [17], and adaptor sequences were removed using Cutadapt v4.4 [18]. Only reads of 18–30 nucleotides were retained, and the 3′ bases with a quality score below 20 were trimmed. Trimmed data were further processed to collect reads aligned with non-coding RNA sequences using Bowtie v1.1.2 [19]. miRNA prediction was conducted using the miRkwood tool [20] with a score range of 0 to 5 alongside BrumiR v3.0 [21]. To quantify miRNA expression levels, clean miRNA-seq reads were mapped to the predicted miRNAs, which were subsequently compared against the miRBase database [22] for validation. Predicted miRNA families were further identified using BLASTn v2.2.29 in the NCBI-BLAST package [23].

### 2.14. miRNA Transfection

Cell lines were transfected with 20 nM miRNA mimic using Lipofectamine RNAiMAX Transfection Reagent (Invitrogen, Carlsbad, CA, USA) following the manufacturer’s protocol. After transfection, the transfected cells or cultured medium were used in other experiments.

### 2.15. Statistical Analysis

All experiments were performed at least 3 times, and data are presented as the mean ± standard deviation (SD). Statistical significance was determined using ordinary one-way ANOVA, followed by Student’s *t*-test, with a threshold of *p* < 0.05 considered statistically significant. All reported data represent the mean ± standard error (SE) unless stated otherwise.

## 3. Results

### 3.1. Isolation and Characterization of the EVs from the Callus of C. japonica L.

**Figure 1:**
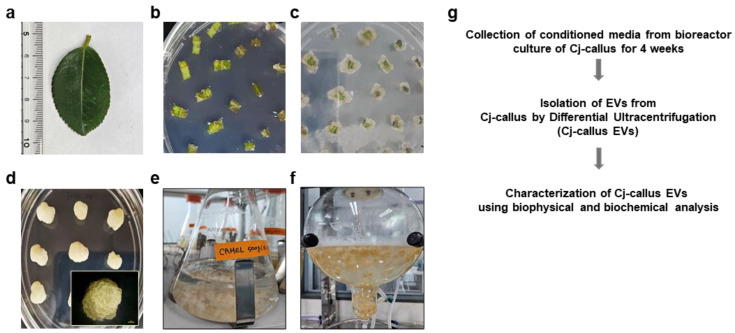
Experimental design for the isolation of EVs derived from *C. japonica* L. callus. (**a**) Image of *Camellia japonica* L. leaf grown in Yeosu, Republic of Korea. (**b**), Sterilized leaf sections with ethanol and chlorox, and then leaf explants were cultured on CIM for callus induction. (**c**) Initiating callus formation from the leaf explants. (**d**) Homogeneous callus cultures on a Petri dish, and the enlarged callus is observed under a microscope, scale bar, 1 mm. (**e**,**f**) Suspension culture of Cj-callus and bioreactor culture. (**g**) Short workflow planned for the purification and characterization steps of EVs released by Cj-callus (Cj-callus EVs).

Initial observations indicated that the explants exhibited swelling and active cell proliferation, particularly along the cut edges, leading to callus formation within 2–3 weeks (Figure 1a–c). Optimal callus induction, as measured by callus size, callus weight, and the percentage of explants producing calli, was achieved using a medium containing 0.3 mg/L 2,4-D. To obtain homogeneous calli, explants were cultured on callus induction medium (CIM), which included basic MS salts with 0.3 mg/L 2,4-D for 3–4 times at 4-week intervals. A homogeneous callus cell line was obtained, characterized by a bright yellow color, rapid multiplication rate, and a slightly sturdy texture (Figure 1d). Callus clusters derived from CIM were subsequently used to initiate suspension cultures. This process involved transferring the callus into a 1 L Erlenmeyer flask containing 400 mL of liquid medium, maintained at 100–300 rpm (Figure 1e). The culture was then serially scaled up by transferring it to a bioreactor with a tenfold volume increase while providing controlled airflow for 4 weeks to ensure optimal growth (Figure 1f). The experimental workflow for isolating and purifying Cj-callus EVs is shown in Figure 1g.

**Figure 2:**
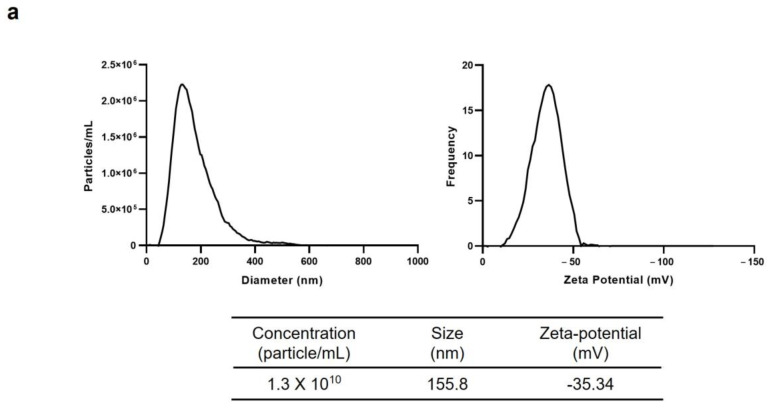

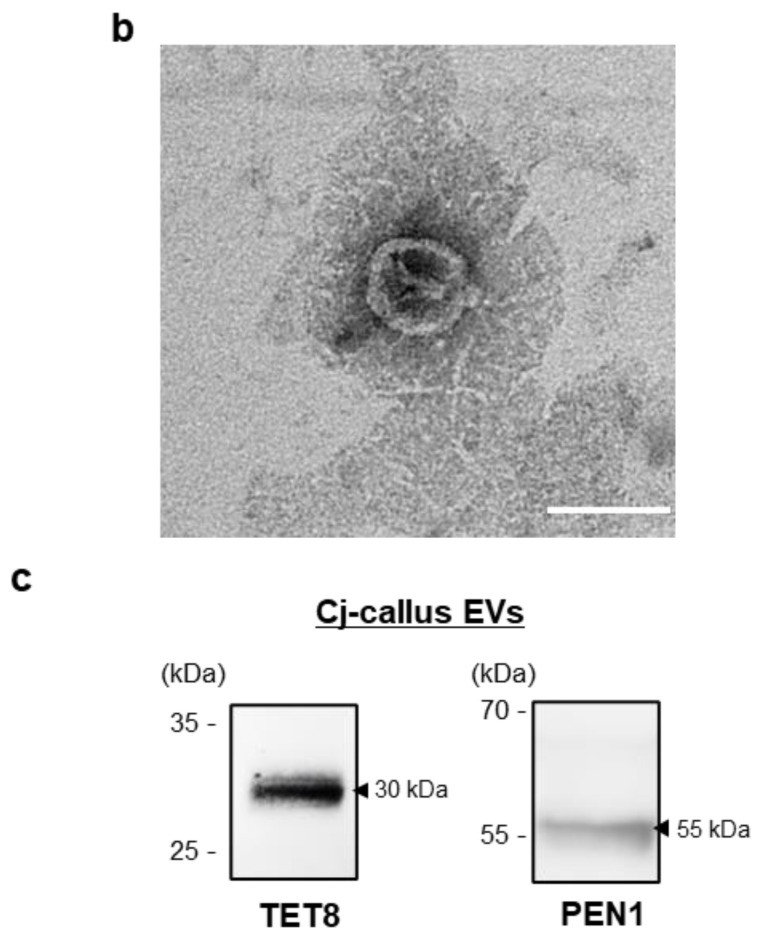
Characterization of Cj-callus EVs. (**a**) Results from NTA of Cj-callus EVs, detailing the size, concentration, and zeta potential. (**b**) An image of Cj-callus EVs was captured by transmission electron microscopy (TEM). Scale bar, 100 nm. (**c**) Western blot result of Cj-callus EVs. 12% SDS-polyacrylamide gels were used. Primary and secondary antibodies were diluted 1:1000 and 1:5000, respectively. See Supplementary for original western Blot images.

The biophysical properties of Cj-callus EVs were assessed by measuring their size, particle concentration, and zeta potential using Nanoparticle Tracking Analysis (NTA). The average particle size, concentration, and zeta potential of the Cj-callus EVs were identified as 155.8 nm, 1.3 × 10^10^ particles/mL, and −35.34 mV, respectively (Figure 2a). Transmission electron microscopy (TEM) further revealed the shape and size characteristics of Cj-callus EVs, confirming their round morphology with a diameter of up to 100 nm (Figure 2b). Western blot analysis was conducted to assess the expression of TET8 and PEN1, which are well-known biomarkers of plant-derived extracellular vesicles from *Arabidopsis thaliana* [24,25]. Bands of TET8 and PEN1 of approximately 30 and 55 kDa, respectively, were successfully detected, indicating the presence of EVs from Cj-callus (Figure 2c).

### 3.2. Enhanced Wound-Healing Activity by Cj-Callus EVs in Skin Cells

**Figure 3:**
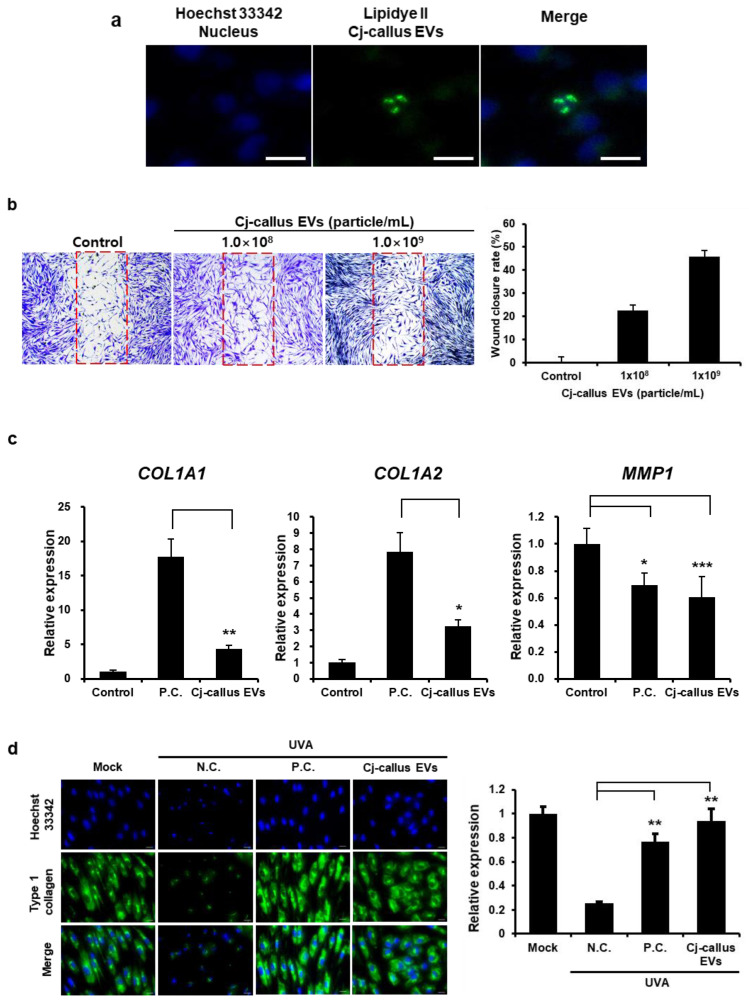
EVs uptake assay and wound healing by Cj-callus EVs. (**a**) LipidyeⅡ-stained EVs’ uptake by keratinocyte. Cj-callus EVs were stained with 1 μM LipidyeⅡ, and the remaining dye was removed using an ultrafiltration membrane. 0.5 μM Hoechst 33342 was used as a counterstain. Scale bar, 10 μm. (**b**) Images of wound-healing activity by EVs in fibroblasts, along with a graph showing the rate of wound closure after EV treatment. The red dotted line indicates the wound area. P.C., Positive Control, 10 ng/mL TGF-β. (**c**) qRT-PCR results of collagen and MMP gene expression after UVA exposure (1.5 J/cm^2^), Cj-callus EVs, 1.0 × 10^9^ particles/mL. * *p* < 0.05; ** *p* < 0.01, *** *p* < 0.001. (**d**) EVs recover type 1 collagen of fibroblasts after UVA exposure. N.C., Negative Control; P.C., Positive Control, 10 ng/mL TGF-β.; Scale bar, 20 μm. ** *p* < 0.01.

To evaluate the biological efficacy of Cj-callus EVs, cell viability assays were performed to determine whether exposure to varying concentrations of Cj-callus EVs influenced the viability of target cells. The results showed no significant differences in cell viability, regardless of the EV concentration (Supplementary Figure S1a). The intracellular uptake of Cj-callus EVs by HaCaT cells was assessed using fluorescence microscopy (Figure 3a). Fluorescently labeled EVs were observed within the cytoplasm, either dispersed or aggregated, indicating successful uptake and the potential for cross-kingdom interactions. To explore the wound-healing potential of Cj-callus EVs, HFFs were treated with Cj-callus EVs. Cj-callus EV treatment significantly accelerated wound closure in a dose-dependent manner, with wound closure rates of 22.6% and 46.0% observed at concentrations of 1.0 × 10^8^ and 1.0 × 10^9^ particles/mL, respectively (Figure 3b).

To determine the effects of Cj-callus EVs on damage repair in UVA-exposed fibroblasts, immunocytochemistry and qRT-PCR were performed to detect collagen and *MMP1* expression. The results showed that *COL1A1* and *COL1A2* expression were significantly upregulated in Cj-callus EV-treated cells, with increases of 4.31-fold and 3.24-fold, respectively, compared to the control. *MMP1* expression was downregulated by 39.29%, similar to the positive controls (10 ng/mL TGF-β) (Figure 3c). Immunocytochemistry was performed to examine the expression level of type I collagen involved in wound healing. Collagen expression in HFF decreased following UVA exposure; however, treatment with Cj-callus EVs restored type I collagen expression, showing a 30.85% increase compared to the negative control at a concentration of 1.0 × 10^9^ particles/mL (Figure 3d).

These results suggest that Cj-callus EVs penetrate the cytoplasm of mammalian cells and exert a concentration-dependent wound-healing effect.

### 3.3. Anti-Inflammatory Activity by Cj-Callus EVs in Keratinocyte

**Figure 4:**
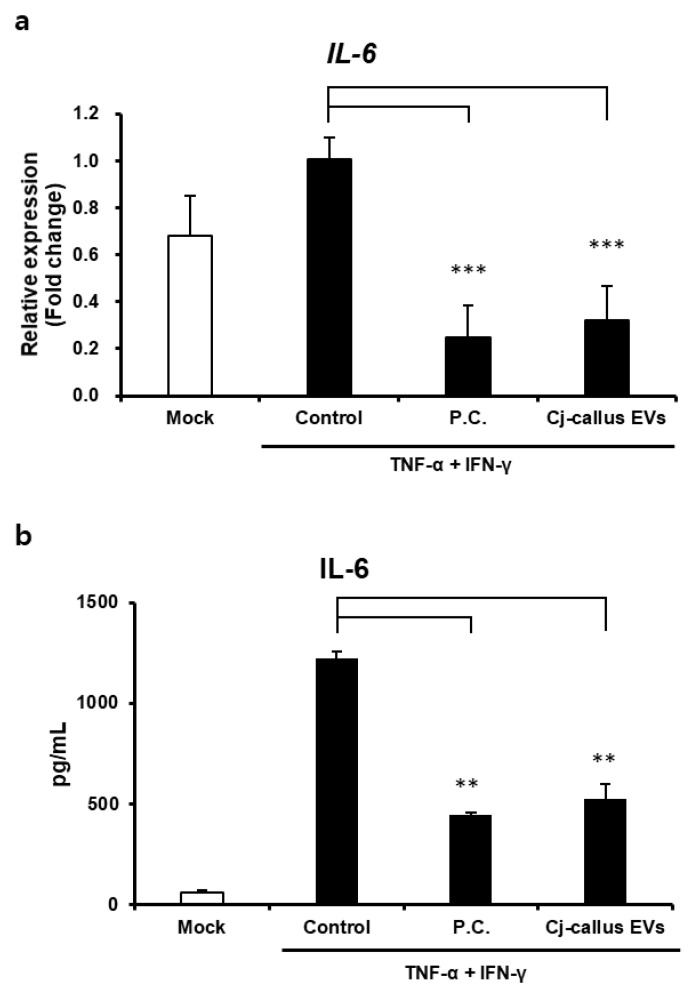
Anti-inflammatory activity of EVs derived from *C. japonica* L. (**a**) Relative expression level of *IL-6* mRNA with EVs. Control, no treatment; P.C., Positive Control, 10 ng/mL TGF-β; *** *p* < 0.001. (**b**) IL-6 protein expression is down-regulated with EVs. ** *p* < 0.01.

Balancing the inflammatory response is essential for optimal wound healing and recovery. Therefore, the expression of IL-6 was investigated to determine the anti-inflammatory activity of Cj-callus EVs because IL-6 plays a crucial role in wound healing by regulating collagen accumulation. To evaluate the anti-inflammatory activity of Cj-callus EVs in human keratinocytes (HaCaT), we measured changes in IL-6 production following treatment with Cj-callus EVs. In HaCaT cells stimulated with TNF-α and IFN-γ, treatment with EVs at a concentration of 1.0 × 10^9^ particles/mL resulted in a 68.21% reduction in *IL6* gene expression (Figure 4a). Furthermore, IL-6 protein production decreased by 57.20% in Cj-callus EV-treated cells compared to that in untreated controls under the same inflammatory conditions (Figure 4b).

These results suggested that Cj-callus EVs have significant anti-inflammatory effects, indicating their potential role in modulating inflammation and supporting wound healing.

### 3.4. Enrichment of miR408 in Cj-Callus EVs

**Figure 5:**
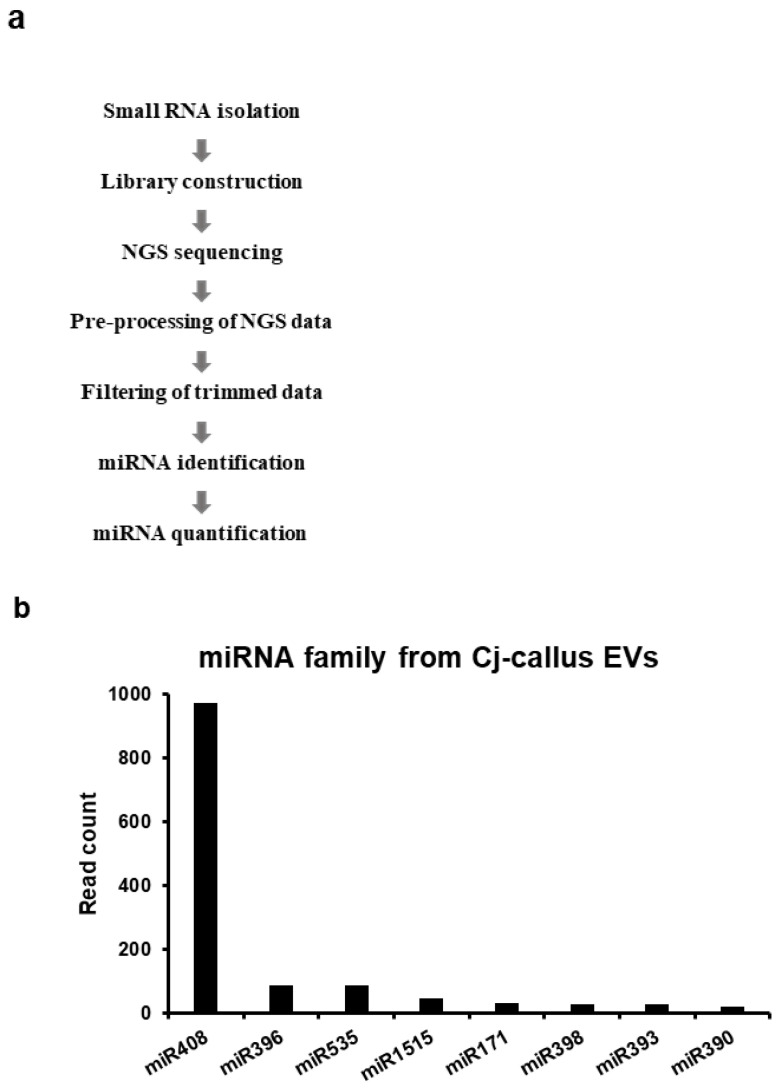
Small RNA sequencing of callus and EVs derived from *C. japonica* L. (**a**) Brief small RNA sequencing flow chart. (**b**) miRNA list from Cj-callus EVs according to read counts. The E-value is a corrected bit-score adjusted to the sequence database size. Blast hits with E-values smaller than 1.0 × 10^−5^ can still be considered as good hits for homology matches.

miRNAs present in EVs serve as key regulators of gene expression and participate in various cellular processes, potentially transmitting these functions to recipient cells. To identify and characterize the miRNAs in Cj-callus EVs, small RNA sequencing was performed on both Cj-callus and Cj-callus EVs derived from the induced calli. This approach enabled the identification and quantification of individual miRNAs. The workflow is summarized in Figure 5a. After trimming the reads to 20–30 nucleotides, the composition of the small RNA categories within the trimmed callus reads was 60.55% ncRNA, 20.42% CDS, 18.83% rRNA, 0.16% tRNA, and 0.04% miRNA. The corresponding composition of the EV reads was 55.93% ncRNA, 32.69% CDS, 10.22% rRNA, 0.73% tRNA, and 0.43% miRNA (Table 1). Notably, the miRNA content in EVs was approximately 1.62 times higher than that in calli, suggesting that EVs selectively enrich and concentrate miRNAs. Although the overall composition ratios of small RNA were similar between the two groups, miRNAs were significantly more abundant in EVs, both in terms of proportion and read count. Further analysis identified eight distinct miRNA families enriched in Cj-callus EVs. Their read counts are detailed in Figure 5b and Supplementary Table S1. These findings suggest the selective enrichment of miRNAs, particularly miR408, suggesting a potential regulatory role of EV-associated miRNAs in various biological functions.

### 3.5. Bioactivity of miR408 in Cj-Callus EVs

**Figure 6:**
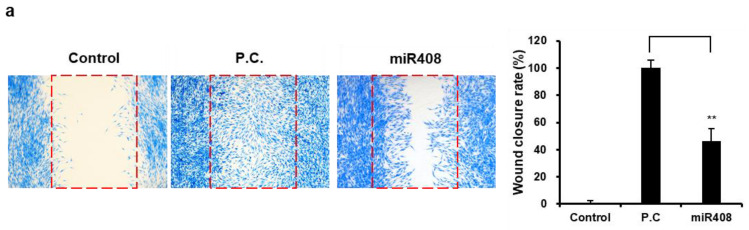

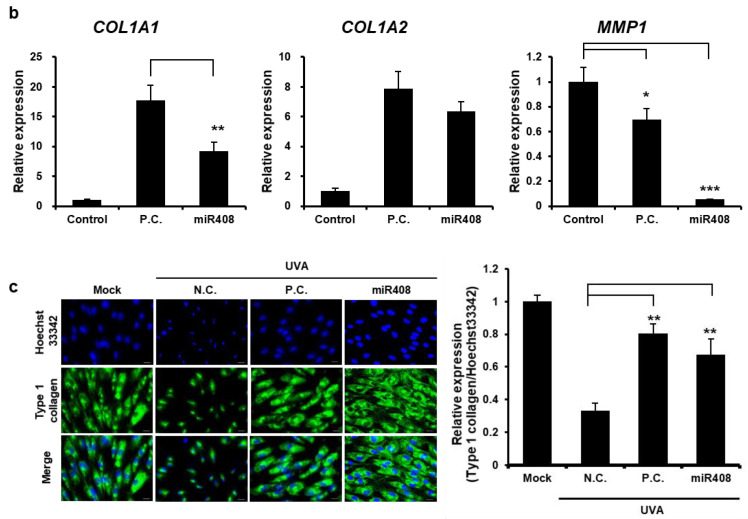
In vitro wound-healing activity of miR408 from Cj-callus EVs. (**a**) Images depicting the wound-healing activity of miR408 in fibroblasts and a graph illustrating the rate of wound closure. The red dotted line indicates the wound area. ** *p* < 0.01. (**b**) qRT-PCR results of collagen and MMP gene expression after UVA exposure. P.C., Positive Control, 10 ng/mL TGF-β. * *p* < 0.05; ** *p* < 0.01, *** *p* < 0.001. (**c**), miR408 restores type I collagen in fibroblasts following UVA exposure. N.C., Negative Control; P.C., Positive Control, 10 ng/mL TGF-β; Scale bar, 20 μm. ** *p* < 0.01.

To explore whether microRNAs derived from EVs exhibit bioactivity similar to that of the EVs themselves, miR408—identified as the most prevalent miRNA within EVs—was chosen for detailed analysis. Although miR408 did not produce notable changes in fibroblast viability (Supplementary Figure S1b), it significantly promoted wound healing, enhancing wound closure in fibroblasts by 46.19% compared to the untreated control group (Figure 6a).

Further investigation focused on miR408’s role in repairing UVA-induced damage in fibroblasts. Through immunocytochemistry and quantitative RT-PCR, the expression of collagen-related genes and *MMP1* was examined. Treatment with 10 nM miR408 led to a dramatic increase in *COL1A1* and *COL1A2* levels—by 9.17-fold and 6.38-fold, respectively. Simultaneously, the expression of *MMP1* was nearly eliminated, showing a reduction of 99.95%, surpassing the suppression achieved by the positive control (Figure 6b).

To assess its effect on type I collagen, another key player in wound healing, immunocytochemistry was performed. After exposure to miR408 at 10 nM, fibroblasts displayed a 34.27% increase in type I collagen compared to the negative control (Figure 6c), suggesting a restorative influence.

**Figure 7:**
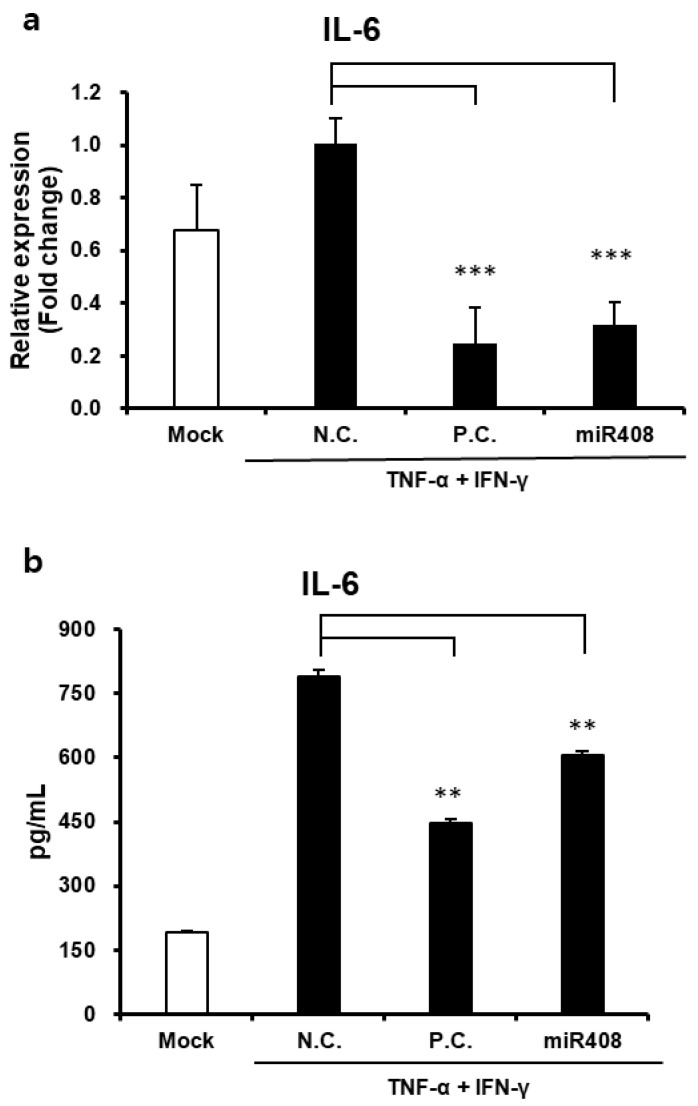
Anti-inflammatory activity of miR408 from EVs. (**a**) miR408 decreases the gene expression of IL-6 from stimulated keratinocytes with TNF-α and IFN-γ. N.C., Negative Control; P.C., Positive Control, 10 ng/mL TGF-β; *** *p* < 0.001. (**b**) IL-6 protein level is reduced with miR408 treatment. ** *p* < 0.01.

To investigate the potential anti-inflammatory properties of miR408 in HaCaT, alterations in IL-6 production were measured following treatment. Administering 10 nM of miR408 led to a 68.21% decline in *IL6* gene expression (Figure 7a). Furthermore, the corresponding IL-6 protein levels were reduced by 57.20% compared to untreated cells exposed to identical inflammatory stimuli (Figure 7b), underscoring miR408′s notable role in suppressing inflammation.

These findings indicate that miR408-derived plant-EVs exhibit substantial anti-inflammatory properties similar to those of EVs, suggesting a promising role in inflammation modulation and wound healing support.

## 4. Discussion

*Camellia japonica* L., commonly known as Japanese camellia or Tsubaki, is highly valued in the cosmetic industry because of its beneficial effects on skin health. The oil extracted from camellia seeds is known for its exceptional moisturizing, hydrating, and skin-nourishing properties [26]. Callus induction and culture from plants, a key technique in plant biotechnology, offer several advantages that make it highly applicable in various fields. One of the primary advantages of callus culture is its ability to efficiently produce large quantities of plant material while ensuring genetic stability. Callus cultures are rich sources of secondary metabolites with diverse applications in medicine, cosmetics, and agriculture [27]. In this study, we utilized plant biotechnology to derive Cj-callus and established a suspension culture using a bioreactor to produce extracellular vesicles (Cj-callus EVs). This system successfully produced EVs from Cj-callus, suggesting the feasibility of harnessing plant-derived EVs as novel bioactive compounds. Our results may indicate the potential of Cj-callus EVs in various sectors, particularly cosmetics, pharmaceuticals, and agriculture, contributing to the expanding research landscape of plant-derived EVs and their diverse applications.

The NTA results demonstrated a uniform distribution of isolated vesicles, as indicated by their consistent zeta potential values, reflecting the homogeneity of the vesicles. This homogeneity suggests their potential for use in drug delivery systems (DDS). Additionally, the successful detection of TET8 and PEN1, which are established EV markers, confirmed that the isolated vesicles were predominantly EVs.

Our findings also revealed that Cj-callus EVs exhibited notable wound healing and anti-inflammatory effects in human-derived cell lines (Figure 3 and Figure 4), supporting their potential for cosmetic and therapeutic applications. In fibroblast cell viability and wound closure assays, treatment with increasing concentrations of Cj-callus EVs resulted in enhanced wound closure, indicating their ability to accelerate healing. This outcome aligns with previous research suggesting that EVs carry bioactive molecules, such as proteins and miRNAs, that can promote cell migration, proliferation, and extracellular matrix (ECM) remodeling during wound healing processes [28]. A key component of the ECM, COL1A1, plays a crucial role in maintaining tissue integrity and structure, especially in connective tissues such as skin, bone, and tendons. Immunocytochemical results suggested that Cj-callus EV treatment restored type I collagen expression, indicating its potential role in ECM reconstruction and skin barrier reinforcement.

In addition to their regenerative properties, Cj-callus EVs exhibit notable anti-inflammatory effects. In human keratinocytes, Cj-callus EV treatment significantly decreased IL-6 expression at both the gene and protein levels in inflammation-induced cells. This anti-inflammatory effect suggests that Cj-callus EVs may modulate key signaling pathways activated by pro-inflammatory cytokines, such as TNF-α and IFN-γ. Although further research is necessary to elucidate the precise mechanisms involved, these findings indicate the potential of Cj-callus EVs to mitigate inflammation-related processes.

Notably, the reduction in IL-6, a cytokine closely associated with inflammation, aging, and skin damage, might indicate the protective role of Cj-callus EVs in managing inflammatory skin conditions. These findings suggest that Cj-callus EVs could serve as promising agents for therapeutic applications targeting skin inflammation, wound healing, and aging.

Research in 2012 discovered that plant-derived miRNAs regulate gene expression in mammals, which sparked significant interest in cross-kingdom miRNA interactions [29]. Inspired by this discovery, we focused on miR408 because of its unique properties and discovered that it exerts a novel effect on improving human skin conditions, suggesting potential benefits beyond cross-kingdom interactions. Small-RNA sequencing confirmed a significantly higher concentration of miRNAs in EVs than in callus cells, indicating selective packaging. The known miRNAs detected were miR408, 396, 535, 1515, 171, 398, 393, and 390, along with several novel miRNAs. The enrichment of miR408 in Cj-callus EVs suggests that miRNAs in plant-derived EVs may serve as functional components capable of transferring regulatory signals to recipient cells.

Our findings suggest the impact of miR408 on human skin, indicating a novel bioactive role that extends across species. miR408 independently enhanced wound healing by promoting wound closure, similar to the effects observed with Cj-callus EVs. *COL1A1* expression is tightly regulated at multiple levels, including transcriptional, post-transcriptional, and post-translational levels. An important mechanism in the post-transcriptional regulation of *COL1A1* involves miRNAs. miR29 families are well-known regulators of COL1A1. Downregulation of miR29 is associated with increased collagen expression and fibrosis in different tissues [30]. In this study, miR408 restored *COL1A1* expression to higher levels than in Cj-callus EVs, with a significant reduction in *MMP1* expression, suggesting that miR408 is involved in collagen formation through MMP-1 inhibition. Additionally, miR408 exhibits notable anti-inflammatory properties by decreasing IL-6 expression in human keratinocytes, similar to the effects of Cj-callus EVs. These results indicate that miR408 plays a crucial role in the wound healing and anti-inflammatory activities of Cj-callus EVs, likely through cross-kingdom regulatory mechanisms. For target gene analysis of miR408, potential genes were predicted using the method described [31]. However, further research is required to explore the correlation between these genes, wound closure, and their anti-inflammatory effects.

## 5. Conclusions

Overall, this study suggests that Cj-callus EVs enriched with functional miR408 have significant therapeutic potential for dermatological applications, particularly in wound healing and inflammation modulation. Future research should focus on elucidating the mechanisms of EV uptake in human skin cells, exploring the pathways modulated by miR408, and assessing the potential of Cj-callus EVs in vivo. This study provides a foundation for the development of plant-derived EV-based formulations and emphasizes the therapeutic relevance of plant-derived EVs in skin health and anti-aging applications.

## Figures and Tables

**Table 1:** Composition of small RNA from Cj-callus and Cj-callus EVs.

		Trimmed Reads	miRNA	rRNA	tRNA	CDS	ncRNA
Cj-callus	Number (#) of Reads	20,070,253	8233	3,779,905	31,977	4,098,468	12,151,670
	Percent (%) of total	100	0.04	18.83	0.16	20.42	60.55
Cj-callus EVs	Number (#) of Reads	3,101,319	13,351	316,953	22,523	1,013,805	1,734,687
	Percent (%) of total	100	0.43	10.22	0.73	32.69	55.93

## Data Availability

The original contributions presented in this study are included in the article. Further inquiries can be directed to the corresponding authors.

## Supplementary Materials

The following supporting information can be downloaded at: https://www.mdpi.com/article/10.3390/biom15081108/s1, Figure S1: Cell viability; Table S1: Small RNA sequencing results; Table S2: Primers used for RT-PCR; Supplementary for original western Blot images.

## References

[1] Rome S. (2019). Biological properties of plant-derived extracellular vesicles. Food Funct..

[2] Zhang H., Freitas D., Kim H.S., Fabijanic K., Li Z., Chen H., Mark M.T., Molina H., Martin A.B., Bojmar L. (2018). Identification of distinct nanoparticles and subsets of extracellular vesicles by asymmetric flow field-flow fractionation. Nat. Cell Biol..

[3] Lian M.Q., Chng W.H., Liang J., Yeo H.Q., Lee C.K., Belaid M., Tollemeto M., Wacker M.G., Czarny B., Pastorin G. (2022). Plant-derived extracellular vesicles: Recent advancements and current challenges on their use for biomedical applications. J. Extracell. Vesicles.

[4] Millar A.A. (2020). The Function of miRNAs in Plants. Plants.

[5] Hill M., Tran N. (2021). miRNA interplay: Mechanisms and consequences in cancer. Dis. Models Mech..

[6] Kim D.H., Park H., Choi Y.J., Im K., Lee C.W., Kim D.S., Pack C.G., Kim H.Y., Choi C.M., Lee J.C. (2023). Identification of exosomal microRNA panel as diagnostic and prognostic biomarker for small cell lung cancer. Biomark. Res..

[7] Lawrie C.H., Gal S., Dunlop H.M., Pushkaran B., Liggins A.P., Pulford K., Banham A.H., Pezzella F., Boultwood J., Wainscoat J.S. (2008). Detection of elevated levels of tumour-associated microRNAs in serum of patients with diffuse large B-cell lymphoma. Br. J. Haematol..

[8] Cui C., Wang J.J., Zhao J.H., Fang Y.Y., He X.F., Guo H.S., Duan C.G. (2020). A Brassica miRNA Regulates Plant Growth and Immunity through Distinct Modes of Action. Mol. Plant.

[9] Pagano L., Rossi R., Paesano L., Marmiroli N., Marmiroli M. (2021). miRNA regulation and stress adaptation in plants. Environ. Exp. Bot..

[10] Gao C., Xu L., Montoya L., Madera M., Hollingsworth J., Chen L., Purdom E., Singan V., Vogel J., Hutmacher R.B. (2022). Co-occurrence networks reveal more complexity than community composition in resistance and resilience of microbial communities. Nat. Commun..

[11] Xiao J., Feng S., Wang X., Long K., Luo Y., Wang Y., Ma J., Tang Q., Jin L., Li X. (2018). Identification of exosome-like nanoparticle-derived microRNAs from 11 edible fruits and vegetables. PeerJ.

[12] Horsburgh S., Fullard N., Roger M., Degnan A., Todryk S., Przyborski S., O’Reilly S. (2017). MicroRNAs in the skin: Role in development, homoeostasis and regeneration. Clin. Sci..

[13] Mathieu E., Benmlih K., Fabre R., Hemmerle J. (2014). An optimized device for staining electron microscopy grids. Biotech. Histochem..

[14] Huang Y., Wang S., Cai Q., Jin H. (2021). Effective methods for isolation and purification of extracellular vesicles from plants. J. Integr. Plant Biol..

[15] Kim C.J., Kuczler M.D., Dong L., Kim J., Amend S.R., Cho Y.K., Pienta K.J. (2022). Extracellular Vesicle Uptake Assay via Confocal Microscope Imaging Analysis. J. Vis. Exp..

[16] Sanada A., Yamada T., Hasegawa S., Ishii Y., Hasebe Y., Iwata Y., Arima M., Sugiura K., Akamatsu H. (2022). Enhanced Type I Collagen Synthesis in Fibroblasts by Dermal Stem/Progenitor Cell-Derived Exosomes. Biol. Pharm. Bull..

[17] Schmieder R., Edwards R. (2011). Quality control and preprocessing of metagenomic datasets. Bioinform.

[18] Martin M. (2011). Cutadapt removes adapter sequences from high-throughput sequencing reads. EMBnet. J..

[19] Langmead B., Trapnell C., Pop M., Salzberg S.L. (2009). Ultrafast and memory-efficient alignment of short DNA sequences to the human genome. Genome Biol..

[20] Guigon I., Legrand S., Berthelot J.F., Bini S., Lanselle D., Benmounah M., Touzet H. (2019). miRkwood: A tool for the reliable identification of microRNAs in plant genomes. BMC Genom..

[21] Moraga C., Sanchez E., Ferrarini M.G., Gutierrez R.A., Vidal E.A., Sagot M.F. (2022). BrumiR: A toolkit for de novo discovery of microRNAs from sRNA-seq data. GigaScience.

[22] Gbriffiths-Jones S., Grocock R.J., van Dongen S., Bateman A., Enright A.J. (2006). miRBase: microRNA sequences, targets and gene nomenclature. Nucleic Acids Res..

[23] Camacho C., Coulouris G., Avagyan V., Ma N., Papadopoulos J., Bealer K., Madden T.L. (2009). BLAST+: Architecture and applications. BMC Bioinform..

[24] He B., Cai Q., Qiao L., Huang C.Y., Wang S., Miao W., Ha T., Wang Y., Jin H. (2021). RNA-binding proteins contribute to small RNA loading in plant extracellular vesicles. Nat. Plants.

[25] Rutter B.D., Innes R.W. (2017). Extracellular Vesicles Isolated from the Leaf Apoplast Carry Stress-Response Proteins. Plant Physiol..

[26] Jung E., Lee J., Baek J., Jung K., Lee J., Huh S., Kim S., Koh J., Park D. (2007). Effect of Camellia japonica oil on human type I procollagen production and skin barrier function. J. Ethnopharmacol..

[27] Efferth T. (2019). Biotechnology applications of plant callus cultures. Engineering.

[28] Narauskaitė D., Vydmantaitė G., Rusteikaitė J., Sampath R., Rudaitytė A., Stašytė G., Aparicio Calvente M.I., Jekabsone A. (2021). Extracellular Vesicles in Skin Wound Healing. Pharmaceuticals.

[29] Zhang L., Hou D., Chen X., Li D., Zhu L., Zhang Y., Li J., Bian Z., Liang X., Cai X. (2012). Exogenous plant MIR168a specifically targets mammalian LDLRAP1: Evidence of cross-kingdom regulation by microRNA. Cell Res..

[30] Cushing L., Kuang P.P., Qian J., Shao F., Wu J., Little F., Thannickal V.J., Cardoso W.V., Lü J. (2011). miR-29 is a major regulator of genes associated with pulmonary fibrosis. Am. J. Respir. Cell Mol. Biol..

[31] Jha N., Mangukia N., Gadhavi H., Patel M., Bhavsar M., Rawal R., Patel S. (2022). Small RNA sequencing and identification of papaya (*Carica papaya* L.) miRNAs with potential cross-kingdom human gene targets. Mol. Genet. Genom..

