# Supplementary material for: miRNA408 from Camellia japonica L. Mediates Cross-Kingdom Regulation in Human Skin Recovery
Source: Biomolecules. 2025 Aug 1;15(8):1108. doi: 10.3390/biom15081108 (PMC12383459; doi:10.3390/biom15081108)
Supplement: Supplementary file 1 [file biomolecules-15-01108-s001.zip › biomolecules-3775210 Supplementary Figure 1; Table 1; Table 2.pdf]

Supplementary Figure 1. Cell viability

a

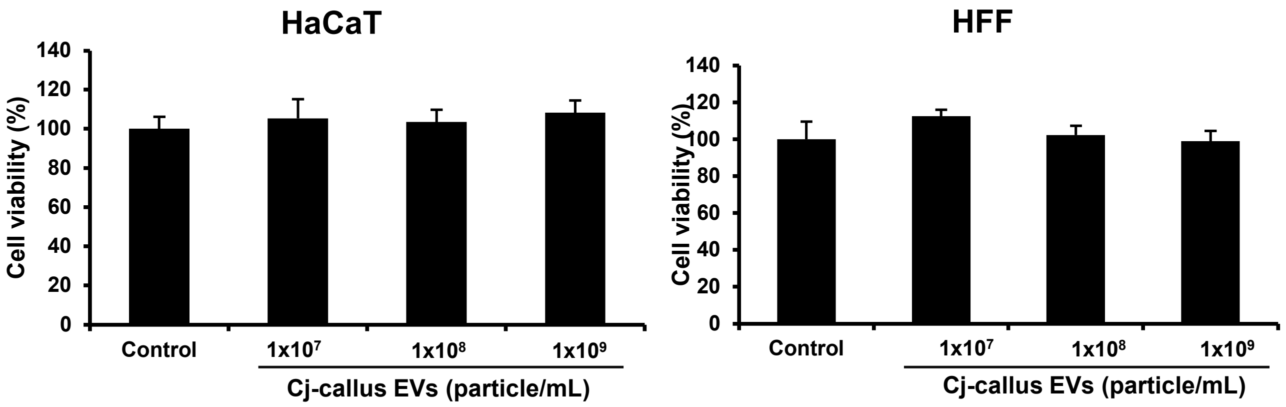

b

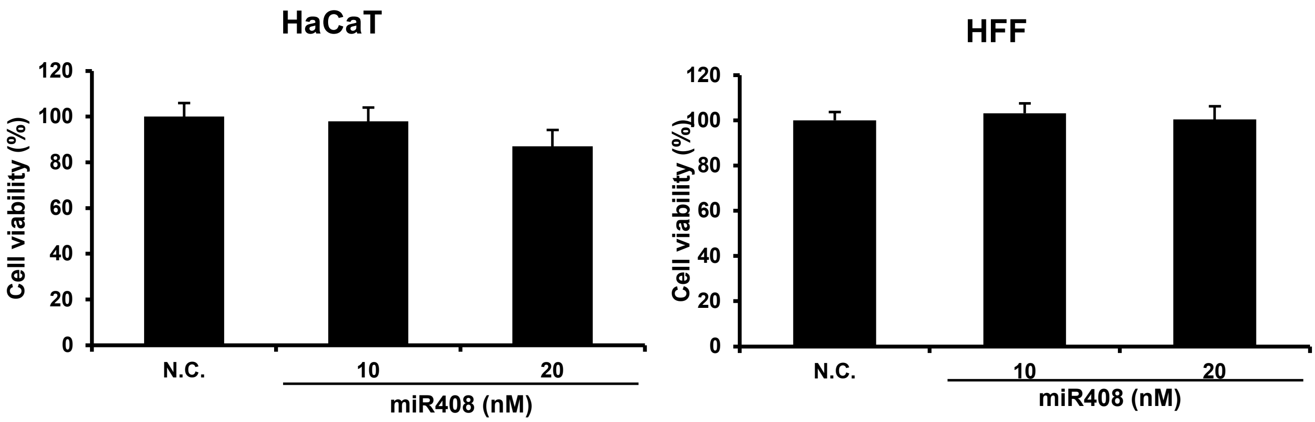

Supplementary Figure 1. Cell viability by EVs and miR408
a, Cell viability of used cell lines(macrophage, fibroblast and keratinocyte) by Cj-callus EVs
b, Cell viability of used cell lines(macrophage, fibroblast and keratinocyte) by miR408

Supplementary Table 1. Small RNA sequencing results

No.	Candidate ID	Sequence	length	Read Count	RPTM	target	miRNA family	qlen	qstart	qend	slen	sstart	send	mis-match	evalue	bitscore
1	Cja_e329	UGACGACGAGAGAGAGCACGC	21	87	280.53	mdm-miR535d	miR535	21	1	21	21	1	21	0	2.00E-07	42.1
2	Cja_e1863	CAAACUUUCAAAACUCAAUU	20	29	93.51	cas-miR857	Novel	20	2	10	22	1	9	0	2.10E+00	18.3
3	Cja_e2153	UGAUGAAGAUCAAGAUGAGGAG	22	22	70.94	ppe-miR6258	Novel	22	6	15	21	12	21	0	6.40E-01	20.3
4	Cja_e2336	ACUGGCUCAGCUUUCUUGGAAC	22	50	161.22	ppe-miR396a	Novel	22	8	18	21	6	16	0	1.60E-01	22.3
5	Cja_e2769	AAUGUAGGUCCACUUUUGUUUG	22	20	64.49	csi-miR159c-5p	Novel	22	7	15	20	12	20	0	2.50E+00	18.3
6	Cja_e2941	GAUUUCAUUCAAAGUCAAGGUG	22	20	64.49	lja-miR11110b-3p	Novel	22	5	14	21	2	11	0	6.40E-01	20.3
7	Cja_e3053	UGAUUGAGCCGUGCCAAUAUC	21	33	106.41	osa-miR171b	miR171	21	1	21	21	1	21	0	2.00E-07	42.1
8	Cja_e3384	CUCUCGAGGCCUCGACAGGUG	21	26	83.84	atr-miR8591	Novel	21	3	11	24	12	20	0	2.30E+00	18.3
9	Cja_e3386	CUCUGGUCCGUAUCUUGUGGU	21	21	67.71	ath-miR5024-3p	Novel	21	8	17	21	1	10	0	5.90E-01	20.3
10	Cja_e3409	AGGGAGACAUGCAGUUGCCAG	21	37	119.30	fve-miR11309	Novel	21	8	17	21	10	19	0	5.90E-01	20.3
11	Cja_e5101	UGGCAUGGGCGGAGUCGGAAA	21	785	2531.18	osa-miR5072	Novel	21	9	17	22	11	19	0	2.30E+00	18.3
12	Cja_e5259	GUAUUGUUGUAUUGUUGGACU	21	20	64.49	bdi-miR7748a-3p	Novel	21	10	20	24	12	22	0	1.50E-01	22.3
13	Cja_e5370	CGCGUACCAAAAGUAAUAAUG	21	27	87.06	mdm-miR11018	Novel	21	8	16	22	8	16	0	2.30E+00	18.3
14	Cja_e5384	CCCCACACCUGACGCUGAAAG	21	21	67.71	stu-miR6149-5p	Novel	21	4	12	21	7	15	0	2.30E+00	18.3
15	Cja_e5508	UUCAACAUAGUAUCAGAACCA	21	19	61.26	osa-miR1873	Novel	21	2	17	24	1	16	1	3.80E-02	24.3
16	Cja_e5787	AAACUAUAAUCUUGAUCUGUGU	22	22	70.94	ata-miR172a-3p	Novel	22	8	16	21	3	11	0	2.50E+00	18.3
17	Cja_e6041	ACGUACACUGCUGUUCCCCUU	21	25	80.61	ath-miR416	Novel	21	1	10	21	8	17	0	5.90E-01	20.3
18	Cja_e6593	CAUGUUAUAGAUUCAGGGUUAG	22	23	74.16	ppe-miR6291c-5p	Novel	22	5	13	21	7	15	0	2.50E+00	18.3
19	Cja_e6604	UCCUUCGCUCAAUAGAGACUCC	22	69	222.49	aof-miR12165	Novel	22	5	13	21	10	18	0	2.50E+00	18.3
20	Cja_e6696	CGAGGGGAAGUAUGAAAAUCC	21	20	64.49	stu-miR8001b-5p	Novel	21	9	19	24	10	20	0	1.50E-01	22.3
21	Cja_e6742	CUUCCCAAAACCUCUCCAUCC	21	32	103.18	gra-miR482c	Novel	21	2	14	22	1	13	0	1.00E-02	26.3
22	Cja_e6880	AACCUAUCCUGAAUUACUUGAA	22	37	119.30	osa-miR5337a	Novel	22	12	20	21	2	10	0	2.50E+00	18.3
23	Cja_e6986	GAUCUUCUUUCUGGUUGCUAAU	22	26	83.84	ath-miR5643a	Novel	22	10	18	21	13	21	0	2.50E+00	18.3
24	Cja_e7092	UCUUUGUUAAUUUUCUAGACU	21	28	90.28	lja-miR7519	Novel	21	9	17	22	3	11	0	2.30E+00	18.3
25	Cja_e7123	UGUUCUCUUUGCUUUCCCCUGA	22	20	64.49	smo-miR1096	Novel	22	5	14	21	5	14	0	6.40E-01	20.3
26	Cja_e7220	GACAGGCAGAUCUGGAAAUACC	22	22	70.94	hbr-miR408b	Novel	22	2	10	22	8	16	0	2.50E+00	18.3
27	Cja_e7255	GGUUGGUUUGCAAUUCUUCUCG	22	21	67.71	ptc-miR482d-5p	Novel	22	1	13	22	8	20	0	1.00E-02	26.3
28	Cja_e7271	GCAGGCGAGCAGUGGUUUGACG	22	23	74.16	zma-miR162-5p	Novel	22	9	18	21	5	14	0	6.40E-01	20.3
29	Cja_e7387	GCCUUUCAAACUUUUUCUCUC	21	22	70.94	osa-miR1435	Novel	21	6	16	20	10	20	0	1.50E-01	22.3
30	Cja_e7752	UGGAGGCGGAUCUGAUCUGGA	21	23	74.16	bra-miR5725	Novel	21	10	19	21	11	20	0	5.90E-01	20.3
31	Cja_e7975	GAGUUGUGCGUGAAUUUAACC	21	23	74.16	csi-miR403b-5p	miR403	21	4	21	21	4	21	1	2.00E-03	28.2
32	Cja_e8139	CCUCAAUUUCAUCUAUAAAUGG	22	19	61.26	lja-miR11077d-3p	Novel	22	1	10	23	10	19	0	6.40E-01	20.3
33	Cja_e8359	AGAUGUUUUUGUAUGAACGGU	21	26	83.84	seu-miR11034	Novel	21	8	16	22	8	16	0	2.30E+00	18.3
34	Cja_e8623	CUUCAACUAGAUCUUGAGCUU	21	20	64.49	hvu-miR6200	Novel	21	4	14	21	7	17	0	1.50E-01	22.3
35	Cja_e8728	GGAGUACAAUGUGAUGAAAUU	21	24	77.39	ppe-miR8125	Novel	21	8	17	21	9	18	0	5.90E-01	20.3
36	Cja_e8750	CUAUUGUGCUUCUGUUUUUUU	21	22	70.94	csi-miR156e-3p	Novel	21	5	15	22	7	17	0	1.50E-01	22.3
37	Cja_e8899	AGCGCUACUUAGGACACCAGA	21	22	70.94	eun-miR10217-5p	Novel	21	3	12	21	12	21	0	5.90E-01	20.3
38	Cja_e9602	AUUAUUAUUAUUUUAGGAGUCU	22	21	67.71	aof-miR12148	Novel	22	10	19	21	3	12	0	6.40E-01	20.3
39	Cja_e9657	CGCCCGGAAGAGAGUCAAUU	20	25	80.61	fve-miR156j	Novel	20	5	14	21	5	14	0	5.40E-01	20.3
40	Cja_e9945	AUUAAUCAGGUGACGUGGCAAA	22	20	64.49	gra-miR8755	Novel	22	11	20	24	15	24	0	6.40E-01	20.3
41	Cja_e10210	AAAAUCAAACUUGGGAUCUCG	21	20	64.49	lja-miR11155c-3p	Novel	21	1	11	24	12	22	0	1.50E-01	22.3
42	Cja_e10283	AUUCCACUCCUCGGACUCUCAU	22	21	67.71	gra-miR8727	Novel	22	12	21	23	1	10	0	6.40E-01	20.3
43	Cja_e10321	UGAAUGCAAUUAAUCAUCCGG	21	20	64.49	stu-miR8025-5p	Novel	21	4	13	24	12	21	0	5.90E-01	20.3
44	Cja_e10426	GAGGUCGAGCCCCGGCGCCGU	21	20	64.49	eun-miR10218-5p	Novel	21	5	13	21	1	9	0	2.30E+00	18.3
45	Cja_e11026	CAAAUUUUCAUUCAAUCCCAU	21	19	61.26	lja-miR11108p-5p	Novel	21	8	17	24	13	22	0	5.90E-01	20.3
46	Cja_e11076	AGAGAAUGUUAGAAAAUGAUU	21	20	64.49	ppe-miR6288b-3p	Novel	21	9	20	21	5	16	0	3.80E-02	24.3
47	Cja_e11178	CGUUAGGACUAUUGGAUGGCU	21	24	77.39	gma-miR5770b	Novel	21	3	12	21	1	10	0	5.90E-01	20.3
48	Cja_e11646	AAGCUCAGGAGGGAUAGCGCC	21	21	67.71	ath-miR390a-5p	miR390	21	1	21	21	1	21	0	2.00E-07	42.1
49	Cja_e11854	AGGGUAAGAUGAAGAAUUGCG	21	20	64.49	mtr-miR5298a	Novel	21	6	16	24	14	24	0	1.50E-01	22.3
50	Cja_e12147	UUCCCACUCCAACUUGAUCGUG	22	23	74.16	ath-miR8179	Novel	22	11	21	21	11	21	0	1.60E-01	22.3
51	Cja_e12183	UCAGAAUGAAAUAAGUUUGAU	21	23	74.16	pab-miR11553	Novel	21	9	17	21	4	12	0	2.30E+00	18.3
52	Cja_e12350	AUCCUUUCUAUGUCAUGUCAAU	22	32	103.18	ath-miR8170-3p	Novel	22	5	13	21	13	21	0	2.50E+00	18.3
53	Cja_e12732	AGGAGGAGAGCGAGAGUUCAGA	22	31	99.96	pab-miR11461	Novel	22	1	11	21	3	13	0	1.60E-01	22.3
54	Cja_e12993	AUUUUGAGUUUCUGAAGGUCU	21	23	74.16	gma-miR5780d	Novel	21	2	15	22	3	16	0	2.00E-03	28.2
55	Cja_e12997	AAGUGUUACAGUAAGGAAUUG	21	21	67.71	gra-miR8723b	Novel	21	3	15	24	10	22	1	2.30E+00	18.3
56	Cja_e13110	UUUUCAUUUUAAUAAAUAUGGA	22	20	64.49	ghr-miR7493	Novel	22	6	15	24	4	13	0	6.40E-01	20.3
57	Cja_e13168	UUCCCACAGCUUUAUUGAACUG	22	88	283.75	zma-miR396g-5p	miR396	22	2	22	21	1	21	0	2.00E-07	42.1
58	Cja_e13856	UGUUUGACUUGACACGUGGCAC	22	24	77.39	gra-miR8739	Novel	22	10	21	24	12	23	0	4.10E-02	24.3
59	Cja_e13922	CAUUCCAAAGACAAGCCUGAA	21	22	70.94	mdm-miR11000	Novel	21	3	11	22	4	12	0	2.30E+00	18.3
60	Cja_e14366	UGAGAGAAGGAACUGAAAACC	21	23	74.16	ath-miR5640	Novel	21	1	12	21	1	12	0	3.80E-02	24.3
61	Cja_e14664	UUCCUUGCGGCGGCGGUGCUC	21	28	90.28	gma-miR4993	Novel	21	7	18	21	3	14	0	3.80E-02	24.3
62	Cja_e15108	CUUUUCUUCGAAUUGGGUUCU	21	24	77.39	pab-miR11454a	Novel	21	9	17	21	12	20	0	2.30E+00	18.3
63	Cja_e15318	UAUAGUCUGACAUUUAGGUGA	21	23	74.16	gma-miR5374-5p	Novel	21	1	13	21	2	14	0	1.00E-02	26.3
64	Cja_e15416	CAUGCUCUGUCUCGUCCCCGU	21	220	709.38	bdi-miR156d-3p	Novel	21	3	12	22	7	16	0	5.90E-01	20.3
65	Cja_e15523	CGUCCACUCUGCAACCCCUUG	21	20	64.49	pab-miR11522	Novel	21	6	13	21	8	15	0	9.20E+00	16.4
66	Cja_e15660	GUUCAAGCAAGUUUCAAUAAU	21	27	87.06	stu-miR8046-5p	Novel	21	9	18	21	9	18	0	5.90E-01	20.3
67	Cja_e17280	CAAGACAACAAUUCCACCUCU	21	22	70.94	ppe-miR6279	Novel	21	3	16	21	2	15	1	5.90E-01	20.3
68	Cja_e18089	UUGUUACAGUUCUCCCCUUCA	21	19	61.26	csi-miR164b-3p	Novel	21	12	20	21	7	15	0	2.30E+00	18.3
69	Cja_e18137	AUCUUCUUUUAACAUCCCGAG	21	20	64.49	pab-miR11521	Novel	21	8	17	21	12	21	0	5.90E-01	20.3
70	Cja_e18466	UCCAAAGGGAUCGCAUUGAU	20	29	93.51	aof-miR393a	miR393	20	1	20	22	1	20	0	6.00E-07	40.1
71	Cja_e18694	CCCGUGUUCUAAAGAUUUGU	20	52	167.67	ath-miR8170-3p	Novel	20	10	18	21	6	14	0	2.10E+00	18.3
72	Cja_e18755	CAUUCCUCGUACUAGAACAAUU	22	21	67.71	aly-miR831-3p	Novel	22	4	13	22	6	15	0	6.40E-01	20.3
73	Cja_e19073	AAUCAAAGAUUUAGAUUCAGC	21	22	70.94	gra-miR8685	Novel	21	3	12	24	11	20	0	5.90E-01	20.3
74	Cja_e19210	AAUUAUUUAGUUCUCAUGUUG	21	22	70.94	gra-miR8781a	Novel	21	1	10	24	6	15	0	5.90E-01	20.3
75	Cja_e19460	UUUUAUCAAAUUUCCCACAUAG	22	21	67.71	csi-miR482f-3p	Novel	22	11	19	21	2	10	0	2.50E+00	18.3

Supplementary Table 1. Small RNA sequencing results

continued

No.	Candidate ID	Sequence	length	Read Count	RPTM	target	miRNA family	qlen	qstart	qend	slen	sstart	send	mis-match	evalue	bitscore
76	Cja_e19803	AUUUGGAAUCAACUCAUAAUUU	22	20	64.49	gra-miR8668	Novel	22	2	12	20	2	12	0	1.60E-01	22.3
77	Cja_e19814	ACAGCAAGUUGUUCUAUUCAA	21	20	64.49	ata-miR169h-3p	Novel	21	4	15	20	1	12	0	3.80E-02	24.3
78	Cja_e19857	UGCACUGCCUCUUCCCUGCCU	21	971	3130.93	ata-miR408-3p	miR408	21	1	20	20	1	20	0	6.00E-07	40.1
79	Cja_e19932	CUACACUUAGAUCUGAAAGAG	21	30	96.73	ath-miR5014b	Novel	21	2	15	21	6	19	1	5.90E-01	20.3
80	Cja_e19984	AUGUGGAUUGUUGGGCCUUU	20	57	183.79	aof-miR12168	Novel	20	4	13	22	10	19	0	5.40E-01	20.3
81	Cja_e20004	AACGGGAAGAGGAGGAUCGAGG	22	23	74.16	lja-miR11117a-3p	Novel	22	5	13	21	8	16	0	2.5	18.3
82	Cja_e20006	AUUUCGAUUCUAUUUUGUCCCU	22	23	74.16	lja-miR11131-5p	Novel	22	3	12	22	9	18	0	0.64	20.3
83	Cja_e21095	UGAGAUGCUUGCACUCCCUUGA	22	45	145.10	ppt-miR2083-5p	Novel	22	8	17	21	3	12	0	0.64	20.3
84	Cja_e21157	CCUGCAUUGAAUCUGAGUUACU	22	20	64.49	vca-miR10206b-5p	Novel	22	5	14	24	15	24	0	0.64	20.3
85	Cja_e21347	UGCACAACUCUCAAUCCUAAUU	22	22	70.94	aof-miR399b	Novel	22	5	12	21	4	11	0	10	16.4
86	Cja_e21590	AGCUUGGGUUUCGACUUUCC	20	18	58.04	lja-miR11100-5p	Novel	20	4	13	21	5	14	0	0.54	20.3
87	Cja_e21759	GAUGAGAGUGCAGACUUGAAA	21	25	80.61	mtr-miR5213-3p	Novel	21	5	14	21	2	11	0	0.59	20.3
88	Cja_e22166	AAUCACUUUCCUCUGAUAUGG	21	20	64.49	bdi-miR9483a	Novel	21	7	16	24	9	18	0	0.59	20.3
89	Cja_e22361	CAAAUGACAACCGAGCUUGAGU	22	28	90.28	gra-miR8757a	Novel	22	11	19	23	5	13	0	2.5	18.3
90	Cja_e22687	ACAUUAGAAGAUAUGCGGGUA	21	22	70.94	ath-miR5628	Novel	21	7	15	21	9	17	0	2.3	18.3
91	Cja_e22715	UGGUGCCCAUUCCCAGAUUUG	21	21	67.71	aof-miR482c	Novel	21	5	14	22	12	21	0	0.59	20.3
92	Cja_e22909	AUGAAGUUCGAAAUCAACGUUG	22	22	70.94	aly-miR3439-5p	Novel	22	7	17	21	8	18	0	0.16	22.3
93	Cja_e22979	AAUUGAUCACGAUUUAAAUAAG	22	21	67.71	gma-miR9747	Novel	22	11	20	22	9	18	0	0.64	20.3
94	Cja_e23002	AGUUAUGUGAGUUUAAUACUUG	22	23	74.16	lja-miR11097e-5p	Novel	22	8	17	21	1	10	0	0.64	20.3
95	Cja_e23135	UCUCGGGAUAAUGCUCAGUGAG	22	39	125.75	pab-miR11562a	Novel	22	7	15	22	5	13	0	2.5	18.3
96	Cja_e23190	CAGUCAAUGUAUUCCCUCCACC	22	25	80.61	bdi-miR7737-5p	Novel	22	3	13	24	6	16	0	0.16	22.3
97	Cja_e23454	GUGUUCUCAGGUCGCCCCUG	20	29	93.51	lus-miR398f	miR398	20	1	20	21	2	21	0	6.00E-07	40.1
98	Cja_e23876	GGUAUGGGAGGUCUAGGCAAGA	22	1911	6161.89	gma-miR9761	Novel	22	9	17	21	4	12	0	2.5	18.3
99	Cja_e23986	AACAAUUCUCUGUCUUCGUUCG	22	22	70.94	csi-miR399e-5p	Novel	22	3	11	21	4	12	0	2.5	18.3
100	Cja_e23993	UUUCCUAUUCCUCCCAUACCU	21	25	80.61	fve-miR482c	Novel	21	1	17	22	3	19	0	4.00E-05	34.2
101	Cja_e24040	GAGUGCCAGAUAUUCAGAGAG	21	27	87.06	mdm-miR10999a	Novel	21	9	16	21	7	14	0	9.2	16.4
102	Cja_e24133	ACGGAGGAUAGCAUGAGGAAG	21	21	67.71	mtr-miR5259	Novel	21	2	10	21	13	21	0	2.3	18.3
103	Cja_e24177	CAGAUUACCAAAGGACUAUUA	21	23	74.16	gma-miR4399	Novel	21	10	18	22	9	17	0	2.3	18.3
104	Cja_e24554	UAUCAAAUAUCACAUCAACAAU	22	23	74.16	nta-miR827	Novel	22	12	21	21	9	18	0	0.64	20.3
105	Cja_e24768	CUUCCACAGCUUUCUUGAACU	21	1188	3830.63	gma-miR396e	Novel	21	2	21	22	1	20	0	6.00E-07	40.1
106	Cja_e24972	UCAUUUUUGCGUGCAGUGAUCC	22	48	154.77	csi-miR1515a	miR1515	22	1	22	22	1	22	1	1.00E-05	36.2
107	Cja_e25117	GCUGCCGGCGACGUCGUGAGA	21	3651	11772.41	pab-miR4414	Novel	21	1	9	21	2	10	0	2.3	18.3
108	Cja_e25476	AGAGUUUAUAUUGGUGGCGUG	21	21	67.71	lja-miR11173-3p	Novel	21	9	17	24	3	11	0	2.3	18.3
109	Cja_e25596	AGAGUUUGUGCGUGAAUCUAA	21	77	248.28	csi-miR403b-5p	Novel	21	3	21	21	1	19	0	3.00E-06	38.2
110	Cja_e25620	CAUGUCUGGUUGGAGUACAAAA	22	20	64.49	zma-miR166g-5p	Novel	22	3	15	21	8	20	0	0.01	26.3
111	Cja_e25682	CUUGUACUUUCUCUUUUGUAUU	22	21	67.71	bdi-miR7743-3p	Novel	22	13	22	24	7	16	0	0.64	20.3
112	Cja_e25713	AGUCAGAUUGAUGAAUGCAUA	21	47	151.55	mtr-miR5227	Novel	21	5	15	22	12	22	0	0.15	22.3
113	Cja_e25811	UGUUCUCCCAUUCCUUUGCAA	21	21	67.71	ata-miR2118a-5p	Novel	21	4	15	22	10	21	0	0.038	24.3
114	Cja_e25856	GGCCGGCCAUUUCCUUUUUUU	21	20	64.49	pab-miR482q	Novel	21	2	11	22	11	20	0	0.59	20.3
115	Cja_e25866	UCCGGGCACGACGCGGCUCU	20	1	3.22	bdi-miR9499	Novel	20	9	17	21	8	16	0	2.1	18.3
116	Cja_e25883	AGUUUAGUGCUCCAUUAAUUUG	22	21	67.71	aly-miR4236	Novel	22	13	22	21	1	10	0	0.64	20.3
117	Cja_e25887	GCCAUCGCCGCUUCCACUUUUU	22	67	216.04	gma-miR5037a	Novel	22	10	18	22	10	18	0	2.5	18.3
118	Cja_e26032	GACGCUCUUUUUGUUCGUUGU	21	27	87.06	stu-miR8011a-5p	Novel	21	6	15	24	13	22	0	0.59	20.3
119	Cja_e26435	GGGGAAUGAAGCCUGGUCCGA	21	605	1950.78	ata-miR166b-5p	Novel	21	2	21	22	1	20	2	0.038	24.3
120	Cja_e26573	UCCCGACCUGCACCAAGCGA	20	92	296.65	ppt-miR1030j	Novel	20	6	15	21	12	21	0	0.54	20.3
121	Cja_e26783	UCCACCGAAGAUCAGAAGGUU	21	23	74.16	pab-miR11511	miR1151	21	1	19	22	1	19	1	0.0006	30.2
122	Cja_e26794	AAUUAUUUUGUCAGGCAGUUUG	22	22	70.94	mdm-miR11007	Novel	22	1	10	21	6	15	0	0.64	20.3
123	Cja_e27044	GGAUCGAGCUGGGCCAGUCAG	21	19	61.26	bdi-miR7766-5p	Novel	21	9	19	24	5	15	0	0.15	22.3
124	Cja_e27155	ACAUGACACUAACCACAUCAUU	22	22	70.94	stu-miR7993b-5p	Novel	22	10	18	24	7	15	0	2.5	18.3
125	Cja_e27168	AUUCUCGAUUCUUUAGCCAUC	21	20	64.49	pab-miR11472	Novel	21	3	13	21	7	17	0	0.15	22.3
126	Cja_e27542	AAAAACUCAUCCUAUCAUUUGC	22	22	70.94	zma-miR159a-5p	Novel	22	10	19	21	5	14	0	0.64	20.3
127	Cja_e27813	AGCUAUGAUUGUUGUAGAAAUU	22	22	70.94	hbr-miR6483	Novel	22	12	22	22	3	13	0	0.16	22.3
128	Cja_e28006	UCAGUGGCAUUGGAUGAAAGG	21	20	64.49	ptc-miR7839	Novel	21	3	14	20	1	12	0	0.038	24.3
129	Cja_e28067	GUCGUGCGCUCUCCUGUUCAG	21	12	38.69	vvi-miR3627-3p	Novel	21	7	17	21	5	15	0	0.15	22.3
130	Cja_e28153	CUACAUCAGCACCAUCUCUACU	22	27	87.06	aly-miR172b-5p	Novel	22	7	16	21	2	11	0	0.64	20.3
131	Cja_e28216	CUUUCUUCAGUUGGUUCAUCG	21	21	67.71	osa-miR5338	Novel	21	5	16	21	6	17	0	0.038	24.3
132	Cja_e28443	GUCCAUCUUCACUGGUGGUG	20	34	109.63	stu-miR8035	Novel	20	2	11	22	1	10	0	0.54	20.3
133	Cja_e28591	UGCCUUCCACCACUCUUCAUA	21	26	83.84	mdm-miR10995	Novel	21	13	21	22	9	17	0	2.3	18.3
134	Cja_e28818	CUUACUUUGAUUAGCAAAAGAC	22	20	64.49	gra-miR8781b	Novel	22	6	14	24	1	9	0	2.5	18.3
135	Cja_e28882	AACACAUCCAAACAAAAUAUC	21	19	61.26	mdm-miR11011b	Novel	21	5	14	21	7	16	0	0.59	20.3
136	Cja_e28900	CAAACUCUCAAACUCCAAAACU	22	25	80.61	gra-miR8727	Novel	22	4	13	23	4	13	0	0.64	20.3
137	Cja_e28993	AUGCUUUGUCUACAUUUGGGA	21	96	309.55	cas-miR158a	Novel	21	4	14	21	9	19	0	0.15	22.3
138	Cja_e29325	UGCAGUUCAGAUAAUGCUGGC	21	31	99.96	gma-miR10439	Novel	21	8	18	21	5	15	0	0.15	22.3
139	Cja_e29616	AACAAGUUGUUCUCCAGACUGC	22	20	64.49	ata-miR169h-3p	Novel	22	3	13	20	2	12	0	0.16	22.3
140	Cja_e30113	UGAUUCAGAAGAUGCUUUGGUU	22	23	74.16	ath-miR5654-3p	Novel	22	8	20	23	3	15	0	0.01	26.3
141	Cja_e30254	UUAAUAACUCGAGUUGAAAAGG	22	24	77.39	pab-miR11430	Novel	22	9	18	21	8	17	0	0.64	20.3
142	Cja_e30451	UUUCCAGAAAUGGUCUUGUUG	21	20	64.49	pab-miR11447	Novel	21	1	10	21	4	13	0	0.59	20.3
143	Cja_e30690	AAACAGAUCAGGUAUCCUCUUA	22	20	64.49	vca-miR10206b-5p	Novel	22	3	14	24	3	14	0	0.041	24.3
144	Cja_e31012	CAGACUCCGGUGGAAUGAAGGA	22	25	80.61	mtr-miR5268a	Novel	22	10	20	24	6	16	0	0.16	22.3
145	Cja_e31165	CAAGGAGGAGAACCACAAAAU	21	21	67.71	pab-miR11564a	Novel	21	12	21	21	4	13	0	0.59	20.3
146	Cja_e31343	GUCCUAGAAUACUUUAAAUAU	21	25	80.61	gma-miR10440	Novel	21	8	21	21	8	21	1	0.59	20.3
147	Cja_e31363	AACUAUACAAUCUACUACCUCA	22	45	145.10	ptc-miR6421-5p	Novel	22	6	16	21	6	16	0	0.16	22.3
148	Cja_e31805	UGGAUGCAGAGGUUUAUCGA	20	40	128.98	zma-miR162-5p	Novel	20	6	20	21	5	19	1	0.14	22.3
149	Cja_e31973	UAUUUGUCUUAUAAAAUAGAUG	22	19	61.26	gma-miR10405a	Novel	22	7	16	21	6	15	0	0.64	20.3
150	Cja_e32147	UUAAUAUAUUAACUAGCGUAUU	22	26	83.84	osa-miR1319b	Novel	22	1	11	24	4	14	0	0.16	22.3
151	Cja_e32394	UAACCGUUCAUUUUCUCUUCC	21	28	90.28	osa-miR5509	Novel	21	9	19	21	5	15	0	0.15	22.3
152	Cja_e32522	GGAGUUCUUGGGAGGUUUAGAA	22	21	67.71	sbi-miR6223-5p	Novel	22	5	15	21	1	11	0	0.16	22.3
153	Cja_e32588	AAGUUCGGGUUUGACCAAUGUG	22	25	80.61	csi-miR403a-5p	Novel	22	4	13	21	6	15	0	0.64	20.3
154	Cja_e33197	AGUGAAAGUUCCCCUUCAGGG	21	20	64.49	csi-miR395b-5p	Novel	21	8	15	21	1	8	0	9.2	16.4
155	Cja_e33463	GCUACAUAUACAGAAUUCGUG	21	28	90.28	gma-miR9765	Novel	21	8	19	22	3	14	0	0.038	24.3

Supplementary Table 2. Primers used for RT-PCR

Gene name		Primer sequence (5′ to 3′)	Length (nt)
hGAPDH	F	TGGAAATCCCATCACCATCTTC	22
	R	CGCCCCACTTGATTTTGG	18
hCOL1A1	F	GACCTCAAGATGTGCCACTC	20
	R	CCAGTCTCCATGTTGCAGAA	21
hCOL1A2	F	GGTGAAGTGGGTCTTCCAGG	20
	R	TAAGGCCGTTTGCTCCAGG	19
hMMP1	F	CCCAGCGACTCTAGAAACAC	20
	R	GCCTCCCATCATTCTTCAGG	21
hIL6	F	AGACAGCCACTCACCTCTTCAG	22
	R	TTCTGCCAGTGCCTCTTTGCTG	22
