# Supplementary figures and images for: miRNA408 from Camellia japonica L. Mediates Cross-Kingdom Regulation in Human Skin Recovery
Source: Biomolecules. 2025 Aug 1;15(8):1108. doi: 10.3390/biom15081108 (PMC12383459; doi:10.3390/biom15081108)

## Raw Data for Fig. 2C (western blot)

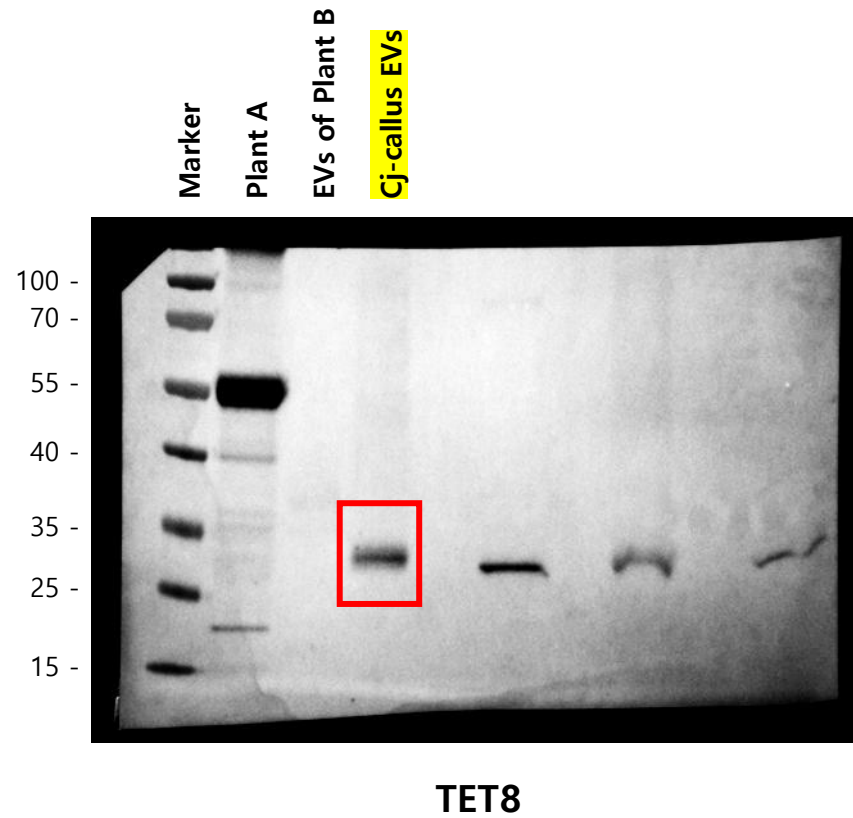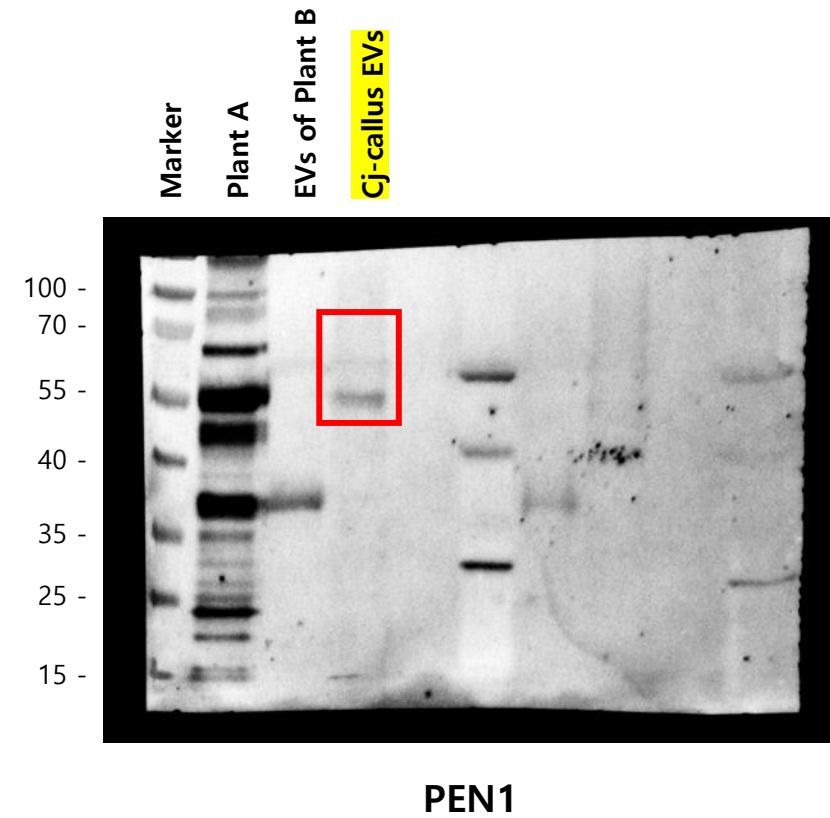

Supplement: Supplementary file 1 [file biomolecules-15-01108-s001.zip › biomolecules-3775210 Supplementary for original western Blot images.pdf]
